# Biatrial Inflammatory and Profibrotic Remodeling in Severe Mitral Regurgitation: A Comparative Tissue and Echocardiographic Study Versus CABG Comparator Group

**DOI:** 10.3390/diagnostics16142183

**Published:** 2026-07-13

**Authors:** Adrian-Grigore Merce, Daniel-Dumitru Nișulescu, Anca Hermenean, Oana-Maria Burciu, Iulia-Raluca Munteanu, Adrian-Petru Merce, Daniel-Miron Brie, Anikó Mornoș, Dragoș Constantin Cozma, Raluca Coifan, Cristian Mornoș

**Affiliations:** 1 Doctoral School Medicine–Pharmacy, “Victor Babeș” University of Medicine and Pharmacy Timișoara, Efimie Murgu Sq. No. 2, 300041 Timișoara, Romania; 2 Department of Cardiology, Institute of Cardiovascular Diseases Timișoara, 300310 Timișoara, Romania; 3 Advanced Research Center, Institute of Cardiovascular Diseases Timișoara, 300310 Timișoara, Romania; 4 Multidisciplinary Doctoral School, “Vasile Goldiș” Western University of Arad, 310025 Arad, Romania; 5 Department of Histology, Faculty of Medicine, “Vasile Goldiș” Western University of Arad, 310025 Arad, Romania; 6 “Aurel Ardelean” Institute of Life Sciences, “Vasile Goldiș” Western University of Arad, 310025 Arad, Romania; 7 Discipline of Radiology and Medical Imaging, “Victor Babeș” University of Medicine and Pharmacy Timișoara, Eftimie Murgu Sq. No. 2, 300041 Timișoara, Romania; 8 Department of Cardiovascular Surgery, Institute of Cardiovascular Diseases Timișoara, 300310 Timișoara, Romania; 9 Cardiology Department, “Victor Babeș” University of Medicine and Pharmacy Timișoara, Eftimie Murgu Sq. No. 2, 300041 Timișoara, Romania

**Keywords:** severe mitral regurgitation, atrial fibrosis, biatrial remodeling, inflammatory biomarkers, interleukin-6, tumor necrosis factor-alpha, transforming growth factor-beta, echocardiography, coronary artery bypass grafting, cardiac surgery

## Abstract

**Background/Objectives:** Severe mitral regurgitation (MR) is associated with chronic atrial stretch, chamber enlargement, pulmonary pressure elevation, and atrial fibrosis, yet the relationship between tissue inflammatory/profibrotic signaling, histologically quantified fibrosis, and echocardiographic remodeling remains incompletely characterized. This study aimed to compare biatrial tissue remodeling in patients with severe MR undergoing mitral valve surgery with a practical non-valvular surgical comparator group undergoing isolated coronary artery bypass grafting (CABG). **Methods:** This single-center, observational, cross-sectional comparative study included 36 elective cardiac-surgery patients: 22 with severe MR and 14 undergoing isolated CABG without significant valvular disease. Left- and right-atrial tissue samples were collected intraoperatively. IL-6, TNF-α, and TGF-β expression was assessed by quantitative real-time PCR using pooled atrial samples stratified by atrial side and study group, whereas atrial fibrosis was quantified histologically on individual tissue specimens using Masson’s trichrome staining and digital image analysis. Clinical, laboratory, and echocardiographic parameters were compared between groups, and exploratory associations were assessed with cautious interpretation. **Results:** Compared with the CABG comparator group, patients with severe MR showed a pooled molecular profile compatible with higher aggregate atrial expression of IL-6, TNF-α, and TGF-β; because qPCR was performed on pooled tissue preparations, these molecular findings were interpreted descriptively and were not used for patient-level inferential statistics. Histologically quantified fibrosis was significantly increased in severe MR in both the left atrium (29.69 ± 12.26% vs. 12.17 ± 4.56%, *p* < 0.0001) and the right atrium (25.25 ± 11.33% vs. 9.01 ± 4.46%, *p* < 0.0001). The MR group also showed more pronounced echocardiographic remodeling, including larger estimated left atrial volume, higher pulmonary artery systolic pressure, and greater right ventricular diameter. Exploratory individual-level analyses were restricted to histological fibrosis and echocardiographic variables. **Conclusions:** Severe MR was associated with marked echocardiographic remodeling and significantly greater histologically quantified biatrial fibrosis compared with a CABG surgical comparator group. Pooled qPCR findings support an aggregate inflammatory/profibrotic signal, but they should be interpreted descriptively because individual-level molecular variability could not be assessed. These findings are hypothesis-generating and do not establish causality.

## 1. Introduction

Mitral regurgitation (MR) is among the most frequent valvular heart diseases in Europe, and echocardiography remains the cornerstone for diagnosis, severity grading, and longitudinal surveillance. In severe primary MR, chronic regurgitant volume overload promotes progressive remodeling of both the left atrium and left ventricle, while guideline-based assessment integrates valve morphology, chamber size, ventricular function, pulmonary pressures, and rhythm status [1].

Left atrial remodeling is particularly relevant in chronic MR because atrial size and volume reflect the cumulative burden of regurgitant load and have prognostic implications. Recent clinical studies have shown that larger left atrial dimensions or left atrial volume index are associated with adverse outcomes in severe MR and with postoperative outcomes after mitral valve repair, supporting the concept that atrial remodeling is not merely an epiphenomenon but an integral component of disease progression [2,3,4,5,6].

Beyond geometric enlargement, severe MR is increasingly recognized as a substrate for atrial fibrosis. Histologic and imaging-based studies have linked advanced atrial remodeling in surgical MR cohorts to fibrosis, endocardial thickening, and impaired atrial mechanics, while experimental work has further supported a causal relationship between MR-related stretch and fibrotic atrial remodeling [7,8,9,10,11,12]. These observations are consistent with the broader concept of atrial cardiomyopathy, in which extracellular matrix expansion, myofibroblast activation, and tissue disorganization contribute to adverse structural and electrical remodeling [13,14].

At the molecular level, inflammatory and profibrotic mediators such as interleukin-6 (IL-6), tumor necrosis factor-alpha (TNF-α), and transforming growth factor-beta (TGF-β) are biologically plausible mediators involved in this process. TGF-β/Smad signaling is regarded as a central profibrotic pathway in atrial remodeling, while inflammatory cascades involving IL-6 and TNF-α have been implicated in atrial fibrosis, arrhythmogenesis, and the progression of atrial fibrillation (AF) [6,8,12,13,14,15,16]. Clinically, AF is highly relevant in this setting because it frequently accompanies atrial remodeling and is associated with important thromboembolic consequences [17,18].

A practical comparative framework for studying MR-related atrial remodeling is represented by patients undergoing isolated coronary artery bypass grafting (CABG) without significant valvular disease. In both groups, intraoperative tissue can be obtained and interpreted alongside echocardiographic data; moreover, echocardiography remains fundamental for defining the cardiac substrate relevant to embolic risk and chamber remodeling [1,4]. However, the extent to which atrial inflammatory and profibrotic signaling can be interpreted together with histologically quantified fibrosis and echocardiographic remodeling within a surgically accessible CABG comparator framework remains insufficiently characterized, particularly with regard to right-atrial involvement. In this context, the present study compared patients with severe MR referred for mitral valve surgery with patients undergoing isolated CABG, focusing on descriptive pooled atrial expression of IL-6, TNF-α, and TGF-β, individual-level histologically quantified fibrosis, and their relationship to echocardiographic indices of remodeling and function.

## 2. Materials and Methods

### 2.1. Study Design and Patient Population

This was a single-center, observational, cross-sectional comparative study performed in patients undergoing elective cardiac surgery at the Institute of Cardiovascular Diseases, Timișoara, Romania. Two groups were included: Group 1 comprised patients with severe mitral regurgitation referred for mitral valve surgery, while Group 2 comprised patients undergoing isolated coronary artery bypass grafting (CABG) for atherosclerotic coronary artery disease, in the absence of significant valvular heart disease. The aim of the study was to compare the clinical profile, echocardiographic parameters, tissue biomarker expression, and degree of atrial fibrosis between the two groups. Only patients with complete clinical, echocardiographic, histological, and molecular data relevant to the present analysis were included. The final study population consisted of 36 patients: 22 patients in Group 1 and 14 patients in Group 2.

### 2.2. Tissue Sampling and Processing

Atrial tissue samples from the auricle were collected intraoperatively under sterile conditions during the surgical procedure. Specimens from both the left atrium and the right atrium were obtained whenever technically feasible and safe. After excision, tissue fragments were divided according to the downstream analyses. One portion was preserved in RNAlater for molecular analysis, one portion was snap-frozen in liquid nitrogen, and one portion was fixed in buffered formalin and embedded in paraffin for histopathological evaluation. Tissue processing and downstream laboratory analyses were performed at Vasile Goldiș Western University of Arad, within the Aurel Ardelean Institute of Life Sciences, following a standardized laboratory workflow to ensure uniformity of tissue handling, preservation, and analysis.

### 2.3. Clinical, Laboratory, and Echocardiographic Assessment

Demographic data, cardiovascular risk factors, comorbidities, rhythm status, previous medical history, and chronic medication were extracted from hospital records and preoperative evaluation files. The analyzed clinical variables included sex, age, residence, anticoagulation status, atrial fibrillation pattern, chronic kidney disease, chronic obstructive pulmonary disease, anemia, dyslipidemia, hypertension, previous myocardial infarction, obesity, heart failure status, and device implantation. Routine laboratory parameters were collected from preoperative blood samples and included hemoglobin, leukocyte count, platelet count, differential leukocyte counts, glucose, creatinine, transaminases, and erythrocyte sedimentation rate. All patients underwent standard transthoracic echocardiography before surgery. The assessed parameters included chamber dimensions, estimated left atrial volume, categorical assessment of atrial dilatation, left ventricular diameters and volumes, left ventricular ejection fraction, transmitral Doppler velocities, E/A ratio, diastolic dysfunction class, pulmonary artery systolic pressure, tricuspid regurgitation gradient, right ventricular function, and the presence and severity of associated valvular lesions. Both continuous and categorical echocardiographic variables were included in the analysis.

### 2.4. RNA Extraction and Quantitative Real-Time PCR

Total RNA was extracted from atrial tissue samples using the Direct-zol RNA MiniPrep Plus kit (Zymo Research, Irvine, CA, USA), according to the manufacturer’s instructions. To ensure adequate RNA yield and analytic stability while preserving a degree of biological replication, atrial specimens were processed as pooled biological preparations rather than as a single pool per study group. For each atrial side and study group, tissue samples were pooled in batches of approximately four patients per pool. When the number of available samples was not divisible by four, the final pool included the remaining available specimens. Accordingly, pooled preparations were generated separately for left-atrial and right-atrial tissue within the severe mitral regurgitation group and the CABG comparator group.

Each pooled preparation was treated as a biological unit for molecular analysis at the pool level. Thus, qPCR-derived variability reflects between-pool variability rather than individual-patient biological variability. RNA concentration and purity were assessed spectrophotometrically. Complementary DNA was synthesized using the High-Capacity cDNA Reverse Transcription Kit (Applied Biosystems, Irvine, CA, USA; 4368814). Quantitative real-time PCR was performed for interleukin-6 (IL-6), tumor necrosis factor-alpha (TNF-α), and transforming growth factor-beta (TGF-β) using SYBR Green Master Mix (Applied Biosystems, Irvine, CA, USA; 4309155) on a StepOnePlus Real-Time PCR System (Applied Biosystems, Irvine, CA, USA). GAPDH was used as the endogenous reference gene, and relative expression was calculated using the 2^−ΔΔCt^ method.

Each pooled preparation was analyzed in technical triplicate. Technical triplicates were used only to assess assay reproducibility and were averaged before statistical analysis. They were not treated as independent biological replicates. Therefore, molecular expression data are presented and interpreted at the pooled-preparation level. These data allow descriptive and exploratory between-group comparison of pooled molecular expression but do not permit direct individual-patient molecular inference.

### 2.5. Histological Assessment of Atrial Fibrosis

Formalin-fixed, paraffin-embedded atrial tissue samples were sectioned at 4–5 μm thickness and stained with Masson’s trichrome for fibrosis assessment. Histological slides were examined by light microscopy using an Olympus BX43 microscope (Olympus, Hamburg, Germany). Digital microscopy images were acquired under standardized magnification and exposure settings and analyzed using ImageJ, 1.54n version software. Whenever available, five randomly selected, non-overlapping fields were analyzed per sample. Fibrosis was expressed as the percentage of collagen-positive area relative to total tissue area and was quantified separately in the left and right atrium. Unlike the pooled molecular workflow, histological fibrosis was treated as a patient-level variable.

### 2.6. Study Endpoints

The primary objective of the study was to compare left and right atrial expression of IL-6, TNF-α, and TGF-β, as well as the degree of atrial fibrosis, between patients with severe mitral regurgitation undergoing mitral valve surgery and CABG patients without significant valvular disease. A secondary descriptive objective was to evaluate pooled left- and right-atrial expression of IL-6, TNF-α, and TGF-β as aggregate molecular signals rather than as individual-level inferential endpoints. Additional secondary objectives were to compare the two groups in terms of clinical and echocardiographic characteristics, treatment profile, and laboratory parameters, and to explore associations between individual-level atrial fibrosis and echocardiographic indices of cardiac remodeling and ventricular function.

### 2.7. Statistical Analysis

Statistical analysis was performed using MedCalc statistical software version 23.4.0. Categorical variables were expressed as absolute numbers and percentages and were compared using the chi-square test or Fisher’s exact test, as appropriate. Continuous variables were expressed as mean ± standard deviation for approximately normally distributed data and as median with interquartile range for non-normally distributed variables. Between-group comparisons were performed using the independent-samples *t* test or the Mann–Whitney U test, depending on data distribution. Molecular qPCR data were analyzed at the pooled-preparation level. For each biomarker and atrial side, technical triplicates were first averaged within each pooled preparation. Summary statistics for IL-6, TNF-α, and TGF-β therefore represent variability across biological pools, not across individual patients. Between-group comparisons of pooled molecular expression were considered exploratory because the unit of analysis was the pool rather than the individual patient.

Histologically quantified fibrosis, clinical variables, laboratory data, and echocardiographic parameters were analyzed at the individual-patient level. Categorical variables were expressed as counts and percentages and compared using the chi-square test or Fisher’s exact test, as appropriate. Continuous variables were expressed as mean ± standard deviation or median [interquartile range], depending on distribution. Between-group comparisons were performed using the independent-samples *t* test, Welch’s *t* test, or the Mann–Whitney U test, as appropriate.

Because molecular expression was measured in pooled preparations, patient-level correlations or regression models using IL-6, TNF-α, or TGF-β as individual-patient variables were not performed. Exploratory correlation and regression analyses were restricted to variables measured at the individual-patient level, particularly histologically quantified fibrosis and echocardiographic parameters. All analyses were interpreted as hypothesis-generating, and a two-sided *p* value < 0.05 was considered statistically significant. All the raw statistical data can be consulted in Supplementary Table S1.

### 2.8. Ethical Considerations

The study was conducted in accordance with the Declaration of Helsinki and local institutional requirements for studies involving human surgical tissue. The doctoral research protocol entitled “Thrombospondins and their impact on cardiac remodeling in atrial myopathy” received ethical approval from the Ethics Committee for Scientific Research of the “Victor Babeș” University of Medicine and Pharmacy Timișoara (approval No. 49/02.10.2023), the Ethics Committee for Research and Development of the Institute of Cardiovascular Diseases Timișoara (approval No. 16027/20.12.2023), and the Ethics Committee for Scientific Research of “Vasile Goldiș” Western University of Arad (approval No. 14/26.03.2024). The approved study sites included the “Victor Babeș” University of Medicine and Pharmacy Timișoara, the Institute of Cardiovascular Diseases Timișoara, the “Aurel Ardelean” Institute of Life Sciences within “Vasile Goldiș” Western University of Arad, and the Faculty of Biology. All participants provided written informed consent before inclusion and tissue sampling.

## 3. Results

### 3.1. Clinical Characteristics and Treatment Profile

The study included 36 patients, of whom 22 were assigned to Group 1 (severe mitral regurgitation referred for mitral valve surgery) and 14 to Group 2 (isolated CABG without significant valvular disease). Age, sex distribution, and area of residence were comparable between groups. Persistent atrial fibrillation was significantly more frequent in Group 1 than in Group 2 (40.9% vs. 7.1%, *p* = 0.0297), whereas postoperative atrial fibrillation did not differ significantly. Baseline demographic and clinical characteristics are summarized in Table 1.

Regarding comorbidity burden, most variables were similarly distributed between groups. However, mixed dyslipidemia was significantly more prevalent in Group 2 (92.9% vs. 59.1%, *p* = 0.0297), and hypertensive/hypertrophic cardiomyopathy was also more frequent in Group 2 (50.0% vs. 18.2%, *p* = 0.0464). No significant differences were observed for chronic kidney disease, anemia, chronic obstructive pulmonary disease, obesity, previous myocardial infarction, or overall hypertension prevalence (Table 2).

**Figure 1:**
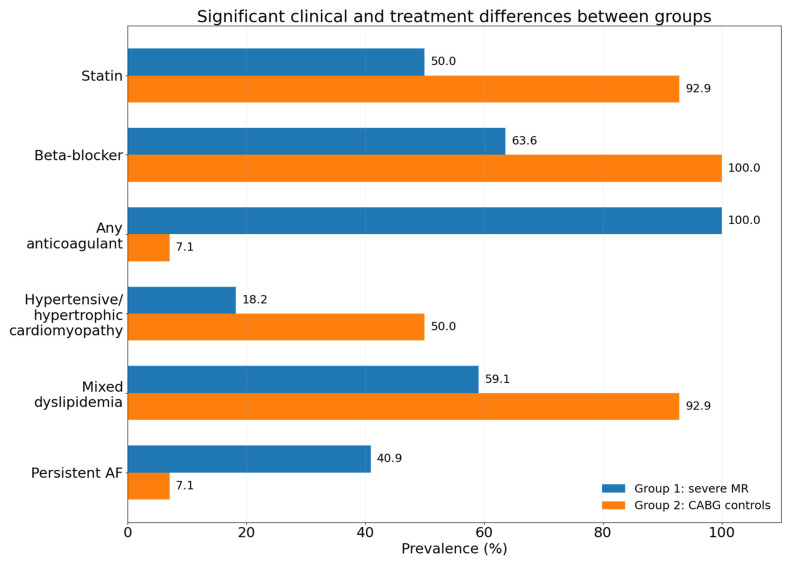
Significant clinical and treatment differences between Group 1 and Group 2. Bar chart summarizing selected categorical variables with statistically significant between-group differences.

With regard to chronic treatment, Group 1 more frequently received anticoagulant therapy overall (100.0% vs. 7.1%, *p* < 0.0001), particularly vitamin K antagonist therapy (100.0% vs. 0.0%, *p* < 0.0001). In contrast, Group 2 more frequently received beta-blockers (100.0% vs. 63.6%, *p* = 0.0116) and statins (92.9% vs. 50.0%, *p* = 0.0087). The use of other cardiovascular drug classes did not differ significantly between groups (Table 3). The most relevant significant differences are summarized visually in Figure 1.

### 3.2. Echocardiographic Findings

Echocardiographic evaluation revealed marked differences in chamber remodeling, ventricular function, diastolic profile, pulmonary pressures, and valvular phenotype between the two groups. Group 1 showed a substantially higher prevalence of left atrial dilatation >4.5 cm compared with Group 2 (100.0% vs. 35.7%, *p* < 0.0001), together with a higher proportion of patients with increased left ventricular end-diastolic diameter >5.7 cm (77.3% vs. 35.7%, *p* = 0.0139). Right atrial dilatation tended to be more frequent in Group 1, although this difference did not reach statistical significance (18.2% vs. 0.0%, *p* = 0.0952). By contrast, atrial dilatation defined using estimated left atrial volume was numerically more frequent in Group 1 but did not differ significantly between groups (95.5% vs. 78.6%, *p* = 0.3042).

Regarding ventricular function and filling pattern, Group 2 more frequently exhibited diastolic dysfunction type I (100.0% vs. 54.5%, *p* = 0.0034), whereas Group 1 showed a higher prevalence of heart failure with preserved ejection fraction (77.3% vs. 42.9%, *p* = 0.0388). Conversely, heart failure with reduced ejection fraction was more common in Group 2 (57.1% vs. 18.2%, *p* = 0.0171). The overall distribution of ejection fraction categories was borderline significant (*p* = 0.0503). These structural and functional findings are summarized in Table 4.

Pulmonary pressure profiles also differed significantly. Mild pulmonary hypertension was more frequent in Group 2 (100.0% vs. 31.8%, *p* = 0.0001), whereas moderate (40.9% vs. 0.0%, *p* = 0.0064) and severe pulmonary hypertension (27.3% vs. 0.0%, *p* = 0.0348) were more prevalent in Group 1. As expected from the surgical indication, Group 1 had universal severe mitral regurgitation, while Group 2 more frequently exhibited lower grades of mitral regurgitation, particularly grade I and grade II. Tricuspid regurgitation grade I was also more common in Group 2 (42.9% vs. 4.5%, *p* = 0.0052). Valvular and pulmonary-pressure-related findings are detailed in Table 5.

Consistent with the operative indication, mitral and tricuspid valve repair procedures, as well as biological and mechanical valve prostheses, were observed predominantly or exclusively in Group 1. These procedure-related findings are shown in Table 6.

**Figure 2:**
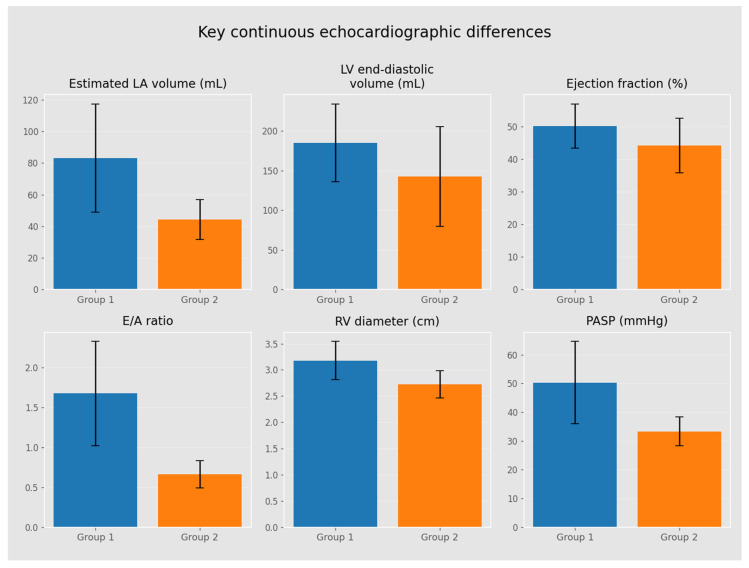
Key continuous echocardiographic differences between groups. Panel summary of selected continuous variables showing the remodeling profile of severe mitral regurgitation versus CABG controls.

Analysis of continuous echocardiographic parameters further supported these categorical differences. Group 1 had a larger left ventricular end-diastolic diameter (5.90 ± 0.74 vs. 5.29 ± 0.79 cm, *p* = 0.0286), higher E/A ratio (1.67 ± 0.65 vs. 0.66 ± 0.17, *p* < 0.0001), higher left ventricular ejection fraction (50.18 ± 6.83 vs. 44.14 ± 8.38%, *p* = 0.0333), larger right ventricular diameter (3.18 ± 0.36 vs. 2.72 ± 0.26 cm, *p* = 0.0001), higher tricuspid peak gradient (44.64 ± 14.21 vs. 27.14 ± 8.23 mmHg, *p* < 0.0001), and higher pulmonary artery systolic pressure (50.32 ± 14.35 vs. 33.29 ± 5.08 mmHg, *p* < 0.0001). Left ventricular end-diastolic volume was also significantly higher in Group 1 (184.86 ± 49.25 vs. 142.43 ± 62.75 mL, *p* = 0.0428). Importantly, estimated left atrial volume was markedly higher in Group 1 than in Group 2 (83.21 ± 34.26 vs. 44.10 ± 12.65 mL, *p* < 0.0001), further supporting the presence of more extensive atrial remodeling in severe mitral regurgitation. These data are shown in Table 7. By contrast, TAPSE was slightly higher in Group 2 (2.21 ± 0.06 vs. 2.09 ± 0.22 cm, *p* = 0.0205), while left ventricular end-systolic volume did not differ significantly between groups. Key continuous echocardiographic contrasts are summarized in Figure 2.

### 3.3. Tissue Biomarker Expression and Atrial Fibrosis

**Figure 3:**
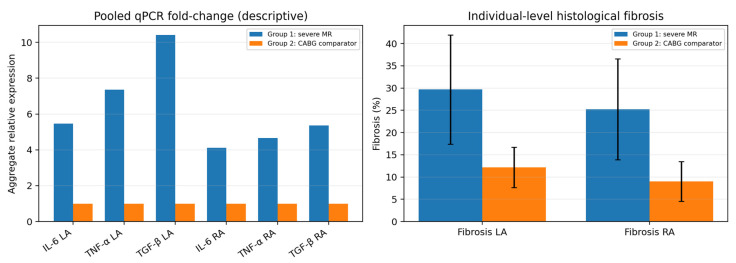
Pooled atrial gene-expression fold-changes and individual-level histological fibrosis in Group 1 and Group 2.

Pooled qPCR analysis suggested a higher aggregate expression profile for IL-6, TNF-α, and TGF-β in both left- and right-atrial tissue from patients with severe MR compared with the CABG surgical comparator group. These molecular results were interpreted descriptively because expression was measured in pooled preparations according to atrial side and study group. Therefore, no biological standard deviations, patient-level *p* values, correlations, or regression analyses were reported for IL-6, TNF-α, or TGF-β expression. Histologically quantified atrial fibrosis, which was measured at the individual-patient level, was markedly increased in Group 1. Left atrial fibrosis was significantly higher in Group 1 than in Group 2 (29.69 ± 12.26% vs. 12.17 ± 4.56%, *p* < 0.0001), and a similar pattern was observed for right atrial fibrosis (25.25 ± 11.33% vs. 9.01 ± 4.46%, *p* < 0.0001). These data are visualized in Table 8. These findings indicate that severe MR is associated with substantially greater biatrial structural remodeling at the histological level, while the pooled molecular data provide descriptive support for an inflammatory and profibrotic tissue signal. These results are visualized in Figure 3.

Pooled qPCR results are shown descriptively as aggregate fold-change values normalized to the CABG comparator group, without biological variability estimates or inferential *p* values. Fibrosis is shown as the mean with standard deviation.

### 3.4. Exploratory Regression Analysis

Exploratory linear regression models were revised to account for the pooled molecular sampling strategy. Because IL-6, TNF-α, and TGF-β were measured in pooled samples, molecular markers were excluded from regression analyses. Models were limited to individual-level variables and were interpreted strictly as hypothesis-generating because of the modest sample size and the lack of adjustment for multiple comparisons.

Within Group 1, no regression model using pooled molecular markers was retained. Given the limited number of patients and the exploratory nature of the analysis, within-group regression modeling was not used to infer mechanistic associations between gene expression, fibrosis, and echocardiographic remodeling.

In the pooled cohort, group assignment itself was significantly associated with the extent of atrial fibrosis and with selected echocardiographic indices. Compared with Group 1, Group 2 was associated with significantly lower left atrial fibrosis (B = −17.52, *p* < 0.0001), lower right atrial fibrosis (B = −16.24, *p* < 0.0001), lower ejection fraction (B = −6.04, *p* = 0.0288), and lower left ventricular end-diastolic volume (B = −42.44, *p* = 0.0380). These models are summarized in Table 9.

## 4. Discussion

The present study suggests that patients with severe mitral regurgitation referred for mitral valve surgery exhibit greater biatrial histological fibrosis, marked echocardiographic remodeling, and a pooled atrial molecular profile compatible with inflammatory and profibrotic activation compared with patients undergoing isolated CABG without significant valvular disease. The principal findings are threefold: first, severe MR was associated with substantially higher individual-level left- and right-atrial fibrosis; second, pooled qPCR analysis suggested higher aggregate atrial expression of IL-6, TNF-α, and TGF-β, although these molecular findings were descriptive only; and third, the MR group showed a more advanced echocardiographic remodeling phenotype involving atrial enlargement, pulmonary pressure elevation, and right-sided chamber changes.

Chronic severe MR imposes sustained volume overload on the left atrium and left ventricle, which promotes chamber dilation, rising wall stress, neurohumoral activation, and progressive extracellular matrix remodeling. This pathophysiologic sequence is well recognized in current valvular heart disease guidance and is increasingly supported by studies demonstrating that left atrial size and volume are clinically meaningful markers of MR severity and prognosis [1,2,3,4,5,6]. In our cohort, patients with severe mitral regurgitation had markedly higher left atrial size parameters, including estimated left atrial volume, which is in line with prior observations that atrial enlargement is an integrative marker of chronic regurgitant burden [2,3,4,5,6].

Our histologic data extend this structural interpretation by showing substantially higher fibrosis in both atria in the MR group. This finding is concordant with previous reports linking severe MR to atrial fibrosis and altered atrial mechanics in surgical populations. Cameli et al. demonstrated that atrial deformation parameters in patients with severe MR undergoing mitral valve surgery predicted left atrial fibrosis and endocardial thickness, while the ALIVE study rationale highlighted atrial fibrosis as a major substrate of adverse remodeling in surgical mitral repair cohorts [7,8]. Experimental work has also reinforced a causal relationship between MR-related mechanical stress and atrial fibrotic remodeling, including porcine and computational MR models in which left atrial fibrosis developed in parallel with molecular pathway activation [9,11].

The pooled molecular profile observed in our study is biologically plausible but must be interpreted cautiously. TGF-β is widely regarded as a central profibrotic mediator in atrial remodeling, acting through canonical Smad-dependent pathways and interacting with fibroblast activation, extracellular matrix deposition, and myofibroblast differentiation. IL-6 and TNF-α are also implicated in atrial inflammation, oxidative stress, endothelial activation, and arrhythmogenic remodeling. However, because gene-expression assays were performed on pooled tissue preparations, the present data cannot determine patient-level variability or prove individual associations between molecular markers and echocardiographic remodeling.

The observed association between MR and atrial fibrosis is also clinically relevant because fibrosis provides a structural substrate for atrial fibrillation. Prior work has shown that MR is associated with left atrial fibrosis in AF populations [10], and broader reviews of atrial fibrosis have consistently identified fibrosis as a hallmark of atrial cardiomyopathy and AF maintenance [12,13,14,15]. In our cohort, persistent AF was more frequent in the severe mitral regurgitation group, which is directionally consistent with this framework. This is important clinically because AF in remodeled atria carries not only rhythm implications but also embolic consequences, as reflected by prior studies on AF-related cardioembolic brain complications and on the inflammatory–metabolic background of AF [17,18].

The echocardiographic phenotype of patients with severe mitral regurgitation also fits the known hemodynamics of severe MR. Patients with MR had larger left atria, larger left ventricular end-diastolic diameter and volume, higher pulmonary pressures, and more advanced valvular abnormalities. Although ejection fraction was higher in patients with severe mitral regurgitation than in the CABG comparator group, this should not be interpreted as indicating less severe disease. In chronic MR, apparent preservation of ejection fraction may coexist with advanced remodeling because part of the stroke volume is ejected into the low-pressure left atrium, whereas the CABG cohort more often reflects ischemic ventricular dysfunction [1]. Recent CMR and longitudinal echocardiographic studies in chronic MR also suggest that myocardial fibrosis and adverse chamber remodeling may already be present despite preserved or seemingly compensated systolic indices, underscoring the limitations of ejection fraction alone as a surrogate of myocardial health [4,5,19].

An additional result deserving specific consideration is the evidence of right-atrial remodeling in severe mitral regurgitation. This pattern is most plausibly explained, at least in substantial part, by the hemodynamic consequences of chronic left-sided volume overload, as reflected in our cohort by higher pulmonary pressures, higher tricuspid gradient, larger right-ventricular diameter, and the greater prevalence of moderate and severe pulmonary hypertension in the MR group. In this framework, right-sided remodeling may represent the downstream consequence of longstanding post-capillary pulmonary hypertension and right-heart load. At the same time, the parallel increase in right-atrial inflammatory and profibrotic markers and in right-atrial fibrosis suggests that the right atrium is not merely a passive bystander, but part of a broader biatrial remodeling response. Therefore, the right-atrial changes observed here are best interpreted as predominantly hemodynamic remodeling, potentially amplified by systemic or chamber-interconnected profibrotic signaling [8,11,12,13,14,15].

The correlation findings were substantially restricted in the revised analysis. Because the molecular measurements were derived from pooled tissue preparations, patient-level correlations involving IL-6, TNF-α, or TGF-β were not retained. The remaining individual-level correlation between right atrial fibrosis and mitral E-wave velocity offers only exploratory context and should not be interpreted as a robust mechanistic relationship, particularly in the absence of multiple-comparison correction.

The absence of retained molecular correlations also limits mechanistic interpretation. The pooled qPCR data support the presence of an aggregate inflammatory/profibrotic signal in severe MR tissue, but they cannot establish whether higher molecular expression occurred in the same patients with greater fibrosis, larger atria, pulmonary hypertension, or more advanced ventricular remodeling. This distinction is important because molecular activation, fibrosis, atrial fibrillation, and hemodynamic overload may coexist without a directly demonstrable individual-level relationship in the present dataset.

Several limitations should be acknowledged. This was a single-center study with a relatively small sample size, limiting statistical power, generalizability, and the feasibility of robust multivariable adjustment or subgroup analysis. The MR and CABG groups were unequal in size and differed in clinically relevant characteristics, including persistent atrial fibrillation, dyslipidemia, cardiomyopathy profile, anticoagulation, beta-blocker use, and statin therapy. Persistent atrial fibrillation is particularly important because it is independently associated with atrial inflammation and fibrosis; therefore, the observed tissue phenotype should be interpreted as associated with the severe MR surgical phenotype rather than as caused exclusively by MR. The CABG group represents a practical non-valvular surgical comparator, not a healthy control group, because coronary artery disease itself may be associated with systemic inflammation, myocardial remodeling, and altered atrial biology [20]. Tissue sampling was also constrained by surgical feasibility and safety, and the exact anatomical sampling site could not be fully standardized, introducing potential sampling-location heterogeneity. The most important molecular limitation is that qPCR analyses were performed on pooled tissue preparations according to atrial side and group. Consequently, molecular expression data were treated as descriptive aggregate fold-change findings only, and patient-level inferential statistics, correlations, or regression analyses involving IL-6, TNF-α, and TGF-β were not considered valid. Finally, several individual-level exploratory analyses were performed without formal correction for multiple comparisons, increasing the possibility of false-positive findings.

In summary, our findings support the concept that severe MR is associated with a broad remodeling phenotype encompassing atrial enlargement, pulmonary pressure elevation, biatrial histological fibrosis, and a descriptive pooled molecular signal compatible with inflammatory and profibrotic activation. Because of the cross-sectional design, the CABG comparator framework, the small sample size, and the pooled qPCR methodology, these findings should be interpreted as hypothesis-generating and not as evidence of causality or patient-level molecular–echocardiographic coupling.

## 5. Conclusions

Patients with severe mitral regurgitation undergoing mitral valve surgery showed significantly greater histologically quantified left- and right-atrial fibrosis and more advanced echocardiographic remodeling compared with CABG patients without significant valvular disease. Pooled qPCR analysis suggested an aggregate atrial molecular profile compatible with inflammatory and profibrotic activation, but these molecular results were descriptive only and could not support patient-level statistical comparisons, correlations, or regression analyses. The CABG group should be interpreted as a practical surgical comparator rather than a healthy control population. Overall, the findings support the presence of extensive biatrial remodeling in severe MR, including right-atrial involvement, but the results remain exploratory and hypothesis-generating because of the small sample size, potential confounding by atrial fibrillation and treatment differences, and the pooled molecular sampling strategy.

## Figures and Tables

**Table 1:** Demographic and baseline clinical characteristics.

Variable	Group 1 (*n* = 22)	Group 2 (*n* = 14)	*p*-Value
Age, years	64.2 ± 9.0	62.4 ± 7.0	0.506
Male sex	14 (63.6%)	11 (78.6%)	0.350
Urban residence	11 (50.0%)	7 (50.0%)	1.000
Home anticoagulation	7 (31.8%)	1 (7.1%)	0.087
Persistent atrial fibrillation	9 (40.9%)	1 (7.1%)	0.030
Postoperative atrial fibrillation	6 (27.3%)	4 (28.6%)	0.933

**Table 2:** Comorbidities and cardiovascular risk profile.

Variable	Group 1 (*n* = 22)	Group 2 (*n* = 14)	*p*-Value
Chronic kidney disease	1 (4.5%)	0 (0.0%)	0.425
Anemia	2 (9.1%)	0 (0.0%)	0.252
Chronic obstructive pulmonary disease	0 (0.0%)	2 (14.3%)	0.072
Dilated cardiomyopathy	13 (59.1%)	5 (35.7%)	0.177
Mixed dyslipidemia	13 (59.1%)	13 (92.9%)	0.030
Fasting impaired glucose	4 (18.2%)	0 (0.0%)	0.095
Hyperuricemia	1 (4.5%)	0 (0.0%)	0.425
Hypertension	19 (86.4%)	13 (92.9%)	0.551
Hypertension grade 1	5 (22.7%)	1 (7.1%)	0.228
Hypertension grade 2	12 (54.5%)	11 (78.6%)	0.149
Hypertension grade 3	2 (9.1%)	1 (7.1%)	0.839
Hypertensive/hypertrophic cardiomyopathy	4 (18.2%)	7 (50.0%)	0.046
Previous myocardial infarction	3 (13.6%)	3 (21.4%)	0.546
Obesity	1 (4.5%)	0 (0.0%)	0.425
Cardiac pacing device	2 (9.1%)	0 (0.0%)	0.252

**Table 3:** Chronic treatment profile.

Treatment	Group 1 (*n* = 22)	Group 2 (*n* = 14)	*p*-Value
Vitamin K antagonist	22 (100.0%)	0 (0.0%)	<0.001
NOAC	0 (0.0%)	1 (7.1%)	0.210
Beta-blocker	14 (63.6%)	14 (100.0%)	0.012
Calcium channel blocker	4 (18.2%)	0 (0.0%)	0.095
Clopidogrel	3 (13.6%)	4 (28.6%)	0.276
Amiodarone	9 (40.9%)	5 (35.7%)	0.759
Diuretic	20 (90.9%)	12 (85.7%)	0.634
Spironolactone	20 (90.9%)	12 (85.7%)	0.634
ARB	4 (18.2%)	1 (7.1%)	0.357
Statin	11 (50.0%)	13 (92.9%)	0.009
Proton pump inhibitor	17 (77.3%)	13 (92.9%)	0.228
Metformin	0 (0.0%)	1 (7.1%)	0.210
SGLT2 inhibitor	2 (9.1%)	3 (21.4%)	0.303
Allopurinol	1 (4.5%)	1 (7.1%)	0.744

**Table 4:** Echocardiographic structural and functional categorical variables.

Variable	Group 1 (*n* = 22)	Group 2 (*n* = 14)	*p*-Value
Right atrial dilatation	4 (18.2%)	0 (0.0%)	0.095
Left atrial dilatation > 4.5 cm	22 (100.0%)	5 (35.7%)	<0.001
Atrial dilatation by estimated LA volume	21 (95.5%)	11 (78.6%)	0.304
LVEDD > 5.7 cm	17 (77.3%)	5 (35.7%)	0.014
LV ejection fraction < 40%	2 (9.1%)	6 (42.9%)	
LV ejection fraction 41–49%	3 (13.6%)	2 (14.3%)	
LV ejection fraction ≥ 50%	17 (77.3%)	6 (42.9%)	0.0503 *
Diastolic dysfunction type I	12 (54.5%)	14 (100.0%)	0.003
Diastolic dysfunction type II	5 (22.7%)	0 (0.0%)	0.058
Diastolic dysfunction type III	5 (22.7%)	0 (0.0%)	0.058
Heart failure	21 (95.5%)	14 (100.0%)	0.425
Heart failure with preserved EF	17 (77.3%)	6 (42.9%)	0.039
Heart failure with reduced EF	4 (18.2%)	8 (57.1%)	0.017

* Global *p* value for comparison across the three EF categories.

**Table 5:** Valvular and pulmonary-pressure-related echocardiographic findings.

Variable	Group 1 (*n* = 22)	Group 2 (*n* = 14)	*p*-Value
Mild pulmonary hypertension	7 (31.8%)	14 (100.0%)	<0.001
Moderate pulmonary hypertension	9 (40.9%)	0 (0.0%)	0.006
Severe pulmonary hypertension	6 (27.3%)	0 (0.0%)	0.035
Aortic regurgitation grade I	6 (27.3%)	2 (14.3%)	0.368
Aortic regurgitation grade II	4 (18.2%)	2 (14.3%)	0.763
Mitral regurgitation grade I	0 (0.0%)	3 (21.4%)	0.025
Mitral regurgitation grade II	1 (4.5%)	7 (50.0%)	0.002
Mitral regurgitation grade III	0 (0.0%)	1 (7.1%)	0.210
Mitral regurgitation grade IV	22 (100.0%)	0 (0.0%)	<0.001
Pulmonary regurgitation grade I	2 (9.1%)	0 (0.0%)	0.252
Tricuspid regurgitation grade I	1 (4.5%)	6 (42.9%)	0.005
Tricuspid regurgitation grade II	13 (59.1%)	8 (57.1%)	0.909
Tricuspid regurgitation grade III	4 (18.2%)	0 (0.0%)	0.095
Tricuspid regurgitation grade IV	4 (18.2%)	0 (0.0%)	0.095
Mild mitral stenosis	1 (4.5%)	0 (0.0%)	0.425
Moderate mitral stenosis	1 (4.5%)	0 (0.0%)	0.425
Severe mitral stenosis	1 (4.5%)	0 (0.0%)	0.425
Mild aortic stenosis	2 (9.1%)	1 (7.1%)	0.839
Severe aortic stenosis	1 (4.5%)	0 (0.0%)	0.425
Mitral valve calcification	1 (4.5%)	1 (7.1%)	0.744
Aortic valve fibrosis	11 (50.0%)	10 (71.4%)	0.210

**Table 6:** Operative characteristics.

Variable	Group 1 (*n* = 22)	Group 2 (*n* = 14)	*p*-Value
Bypass associated with valve surgery	3 (13.6%)	0 (0.0%)	0.155
Mitral valve repair	7 (31.8%)	0 (0.0%)	0.020
Tricuspid valve repair	12 (54.5%)	0 (0.0%)	<0.001
Biological prosthetic valve	7 (31.8%)	0 (0.0%)	0.020
Mechanical prosthetic valve	8 (36.4%)	0 (0.0%)	0.012

**Table 7:** Continuous echocardiographic variables.

Variable	Group 1	Group 2	*p*-Value
A mitral	0.863 ± 0.347	0.950 ± 0.145	0.306
Ascending aorta, cm	3.221 ± 0.276	3.411 ± 0.412	0.143
Aortic annulus, cm	2.214 ± 0.160	2.400 ± 0.290	0.042
LVEDD, cm	5.900 ± 0.737	5.293 ± 0.785	0.029
E/A ratio	1.673 ± 0.653	0.663 ± 0.170	<0.001
E mitral	1.963 ± 3.162	0.636 ± 0.213	0.063
Ejection fraction, %	50.182 ± 6.829	44.143 ± 8.384	0.033
Pmax Ao, mmHg	6.155 ± 6.733	5.639 ± 6.338	0.818
Tricuspid peak gradient, mmHg	44.636 ± 14.208	27.143 ± 8.226	<0.001
Pmed Ao, mmHg	4.136 ± 5.321	2.929 ± 3.792	0.433
Posterior wall thickness, cm	1.045 ± 0.106	1.184 ± 0.134	0.003
Pulmonary artery systolic pressure, mmHg	50.318 ± 14.351	33.286 ± 5.075	<0.001
Septal thickness, cm	1.137 ± 0.189	1.228 ± 0.178	0.155
TAPSE, cm	2.086 ± 0.217	2.207 ± 0.062	0.021
Right ventricular diameter, cm	3.178 ± 0.365	2.724 ± 0.261	<0.001
Vmax Ao, m/s	1.155 ± 0.462	1.148 ± 0.433	0.964
LV end-diastolic volume, mL	184.864 ± 49.246	142.429 ± 62.753	0.043
LV end-systolic volume, mL	101.000 ± 34.221	82.000 ± 49.009	0.219
Estimated left atrial volume, mL	83.214 ± 34.259	44.100 ± 12.646	<0.001

**Table 8:** Pooled atrial tissue biomarker expression and individual-level histological fibrosis in Group 1 and Group 2.

Variable	Group 1	Group 2	*p*-Value/Interpretation
IL-6 LA	5.470-fold	1.000 calibrator	Descriptive pooled qPCR
TNF-α LA	7.353-fold	1.000 calibrator	Descriptive pooled qPCR
TGF-β LA	10.418-fold	1.000 calibrator	Descriptive pooled qPCR
Fibrosis LA, %	29.690 ± 12.260	12.173 ± 4.556	<0.001
IL-6 RA	4.115-fold	1.000 calibrator	Descriptive pooled qPCR
TNF-α RA	4.663-fold	1.000 calibrator	Descriptive pooled qPCR
TGF-β RA	5.367-fold	1.000 calibrator	Descriptive pooled qPCR
Fibrosis RA, %	25.248 ± 11.329	9.007 ± 4.462	<0.001

**Table 9:** Exploratory univariate regression models in the pooled cohort based on group assignment.

Dependent Variable	Predictor	B Coefficient	*p*-Value	R^2^
Fibrosis LA	Group 2 vs. Group 1	−17.516	<0.001	0.434
Fibrosis RA	Group 2 vs. Group 1	−16.241	<0.001	0.433
Ejection fraction	Group 2 vs. Group 1	−6.039	0.029	0.142
LV end-diastolic volume	Group 2 vs. Group 1	−42.435	0.038	0.131

## Data Availability

The original contributions presented in this study are included in the Supplementary Material. Further inquiries can be directed to the corresponding author.

## Supplementary Materials

The following supporting information can be downloaded at: https://www.mdpi.com/article/10.3390/diagnostics16142183/s1. Table S1: Raw statistical data.

## Abbreviations

The following abbreviations are used in this manuscript:

AF	atrial fibrillation
ARB	angiotensin receptor blocker
CABG	coronary artery bypass grafting
cDNA	complementary DNA
CI	confidence interval
COPD	chronic obstructive pulmonary disease
EF	ejection fraction
ESR	erythrocyte sedimentation rate
GAPDH	glyceraldehyde-3-phosphate dehydrogenase
HF	heart failure
HFpEF	heart failure with preserved ejection fraction
HFrEF	heart failure with reduced ejection fraction
IL-6	interleukin-6
IQR	interquartile range
LA	left atrium/left atrial
LV	left ventricle/left ventricular
LVEDD	left ventricular end-diastolic diameter
MR	mitral regurgitation
NOAC	non-vitamin K antagonist oral anticoagulant
PASP	pulmonary artery systolic pressure
PCR	polymerase chain reaction
RA	right atrium/right atrial
RNA	ribonucleic acid
RT-qPCR	reverse transcription quantitative polymerase chain reaction
SD	standard deviation
SGLT2	sodium-glucose cotransporter 2
TAPSE	tricuspid annular plane systolic excursion
TGF-β	transforming growth factor-beta
TNF-α	tumor necrosis factor-alpha
VKA	vitamin K antagonist
